# An evaluation of the use of pentosidine as a biomarker for ageing turtles

**DOI:** 10.1093/conphys/cow076

**Published:** 2017-01-27

**Authors:** John B. Iverson, Randal S. Stahl, Carol Furcolow, Fred Kraus

**Affiliations:** 1 Department of Biology, Earlham College, Richmond, IN 47374, USA; 2 APHIS, US Department of Agriculture, 4101 La Porte Avenue, Fort Collins, CO 80521, USA; 3 Department of Ecology and Evolutionary Biology, University of Michigan, Ann Arbor, MI 48109, USA

**Keywords:** Ectotherm, hydroxyproline, Kinosternidae, *Kinosternon flavescens*, oxygen stress

## Abstract

Concentrations of the biomarker pentosidine have been shown to be useful measures of age for a number of avian and mammalian species. However, no study has examined its usefulness as an age marker in a long-lived ectotherm despite the fact that such a marker could prove useful in understanding age distributions of populations subject to conservation programmes. Therefore, we evaluated pentosidine concentrations in the interdigital webbing of 117 female yellow mud turtles (*Kinosternon flavescens*) at a 35 year study site in western Nebraska where nearly all turtles are of known age. Pentosidine concentrations were extraordinarily low and positively correlated with age in this turtle, but concentrations were too variable to permit precise estimates of age for turtles of unknown age. These results may reflect the remarkable physiological adaptations of this turtle to low temperatures and oxygen deprivation in a highly seasonal environment requiring prolonged hibernation. Whether pentosidine concentrations in other ectotherms occupying less seasonal environments would be more highly correlated with age remains to be determined. However, our results suggest that patterns of accumulation of pentosidine in ectotherms may be fundamentally different from those in endotherms.

## Abstract (precis)

Concentrations of the chemical pentosidine in the bodies of endotherms are highly correlated with age, providing a useful biomarker for ageing individuals and potentially for assessing demographic structure in endangered populations. In this first study of pentosidine in an ectotherm, we found only a weak correlation in a turtle.

## Introduction

Turtles (Order Testudines) are among the longest living of all vertebrate groups (Gibbons, 1987), and their life-history traits (e.g. delayed maturity, high adult survivorship, extended iteroparity) have co-evolved with that longevity. Unfortunately, this life-history strategy makes turtles particularly vulnerable to direct (e.g. harvest, accidental mortality) and indirect human impacts (e.g. habitat loss, climate change, invasive predators and competitors). Indeed, more than half of the world's turtle species are threatened with extinction (Turtle Taxonomy Working Group, 2014).

This extended longevity complicates our ability to assess the demographic status of natural populations without long-term mark–recapture studies, which are expensive both in time and money and infeasible for application to conservation programmes that cannot wait decades for demographic data to appear. For many populations, there may not be sufficient time left to undertake such studies before their disappearance. Furthermore, because age and size are not necessarily correlated in turtles (e.g. Congdon *et al*., 2001; Bury *et al*., 2010), turtle populations may appear to be robust, with seemingly high adult densities, but in reality may be heavily skewed toward old individuals and suffering from severe reductions in recruitment. Thus, although a turtle population might appear healthy, its impending extirpation may be inevitable (e.g. Thompson, 1983; Germano and Bury, 2001; Browne and Hecnar, 2007). The ability to construct age-class distributions for natural populations is essential for evaluating their demographic status and for modelling their future survival probability, but current methods for ageing turtles (e.g. scute annuli and skeletochronology) have limited value (Bjorndal *et al*., 1998; Wilson *et al*., 2003).

Pentosidine is a metabolic byproduct of the Maillard reaction, specifically the non-enzymatic glycosylation of collagen (Chaney *et al*., 2003; Cooey *et al*., 2010). Given that it is metabolically stable (Monnier, 1989; Sells and Monnier, 1989) and accumulates in a number of easily accessible tissues throughout the lifetime of an individual, it has been shown to be a useful biomarker for age in a number of domestic and wild bird and mammal species (Sell *et al*., 1996; Fallon *et al*., 2006; Cooey *et al*., 2010; but see Rattiste *et al*., 2015). Hence, the concentration of pentosidine in tissues of living endotherms has shown great promise for ageing other species non-destructively and without long-term monitoring. However, to date, this relationship has not been examined in any ectotherm. We sought to correct this deficiency by examining pentosidine levels in the skin—following the practice of Sells and Monnier (1989), Chaney *et al*. (2003), Cooey *et al*. (2010) and others—of a very long-lived turtle species, the yellow mud turtle (*Kinosternon flavescens*; YMT), to determine whether they correlate with the known ages of these turtles from a long-term (35 year) mark–recapture study at a field site in western Nebraska.

We sought to determine the value of using pentosidine as a non-invasive biomarker for the rapid assessment of age-class distributions in turtles populations and for ageing confiscated animals of threatened species intended for captive-breeding recovery programmes. We expected that the long lifespans of turtles should allow for a sufficient span of time to detect patterns in pentosidine accumulation.

## Materials and methods

### Focal species and tissue collection

Our field site is located in the western Sandhills region of Nebraska on the Crescent Lake National Wildlife Refuge, adjacent to the Gimlet Lake wetland complex (41° 45′ 22″N; 102° 26′ 12″W). Turtles (including the YMT) have been under study at this site by J.B.I. since 1981 (Iverson, 1991; Iverson and Smith, 1993; Iverson *et al*., 1997; Converse *et al*., 2005). Most individual YMTs are now of known age. In addition, those older than 35 years were assigned estimated ages based on counts of growth annuli (Germano and Bury, 1988) when turtles were first caught as juveniles in the early 1980s. Several individuals first caught as adults at that time were given minimal age estimates based on the minimal number of plastral growth annuli present. The annual deposition of an annulus has been confirmed in our population (J.B.I., unpublished observations), although separate annuli cannot be distinguished in years of little or no growth (Iverson, 2001). Hence, some older individuals are known to be older than 55 years (i.e. had at least 20 countable annuli when first captured in 1981). For our work, turtle age is the number of winters post-hatching.

Turtles (117 females ranging in age from 1 to 53 winters) were sampled (primarily at drift fences; see Iverson, 1991 for details) in June 2015 during the nesting season. Skin samples were excised from the interdigital membrane (webbing) of the clawless (lateral) digit of the right hindfoot. Wounds rarely bled, but we closed them with antibacterial skin glue as a precaution against infection. Recaptures of sampled individuals demonstrated rapid healing and no signs of infection. Skin samples were frozen at approximately −15°C in vials of distilled water immediately after removal and remained frozen until analysis.

### Sample extraction and analysis

All samples were processed and analysed within 5 months of collection. Tissue preparation and determination of pentosidine (Ps) and hydroxyproline (HYP) concentrations followed the techniques described below. Hydroxyproline was measured to estimate the collagen content in the tissue sample, reported in milligrams, referenced to a calf-skin collagen sample (Rousselot Peptan B 5000 HD) with a known HYP concentration of 11.5% (see also Chaney *et al*., 2003; Fallon *et al*., 2006; Cooey *et al*., 2010). Hydroxyproline is not expected to vary with age (Maekawa *et al*., 1970). The Ps concentration was determined relative to the mass of collagen estimated to be present in the sample (in picomoles of pentosidine per milligram of collagen).

Samples were extracted in 2 ml of 2:1 chloroform/methanol (MeOH) to remove the lipid fraction (Folch *et al*., 1957; Hamilton *et al*., 1992). The solvent was decanted and the remaining sample dried at 60°C for 10 min after rinsing with 2 ml of MeOH, then acid hydrolysed in 4 ml of 6 N HCl with a CEM Discover SPD microwave digestor. Samples were digested in a 10 ml quartz tube. Temperature was maintained at 185°C for 15 min, after ramping from ambient over 5 min. Samples were exposed to a maximal microwave energy of 300 W and digested at a maximal pressure of 200 psi. Samples were continuously stirred with a magnetic stir bar during digestion. The acid hydrolysate was dried under vacuum at 60°C with a Rotovap. The sample was reconstituted in 1.0 ml of 25% MeOH in water containing 0.1% heptafluorobutyric acid.

Pentosidine was determined in the hydrosylate using a high-performance liquid chromatography method based on that of Bank *et al*. (1997) following centrifuge filtration of the extract using a Durapore Polyvinylidene fluoride 0.45 μm filter (Millipore). Chromatography was performed using an Agilent 1100 Liquid Chromatograph with a fluorescence detector. A 3 μl sample was injected onto an Agilent XDB-C8 3 mm × 150 mm, 3.5 μm column. Pentosidine was eluted in a gradient starting at 90%/10% water/MeOH and ramping to 75%/25% water/MeOH containing 0.1% heptafluorobutyric acid at 11 min at a constant flow rate of 0.9 ml/min. The MeOH was ramped to 60% at 8.4 min and then to 100% at 13 min and held constant for 4 min to flush the column. The column was re-equilibrated for 3 min before the next injection. The column was maintained at 60.0°C. Pentosidine was detected by fluorescence, with excitation at 328 nm and monitoring for fluorescence emission at 378 nm. The pentosidine concentration was determined using an external standard curve covering the concentration range of 0–25 pmol/ml prepared from a primary standard acquired from Polypeptide (Strasbourg, France). Pentosidine eluted at ~8.9 min in these chromatographic conditions. The pentosidine concentration is reported in picomoles per milligram of collagen.

The collagen content in the tissues was estimated by determining the concentration of HYP in the hydrosylate. Hydroxyproline determination was based on the secondary-amine derivatization method of Hutson *et al*. (2003). To summarize this method, primary amines are derivitized with o-pthalaldehyde, and then secondary amines are derivitized with fluorenylmethyloxycarbonyl chloride. We adapted this method by diluting 0.05 ml of our extract with 0.2 ml of 0.25 M boric acid, pH 9.5, and 0.05 ml of a 200 ppm sarcosine internal standard in 0.70 ml of water. We followed the rest of the derivitization procedure as published. Concentrations were determined with an external standard curve over the range of 0–25.0 ppm HYP using a primary standard (Sigma-Aldrich). Concentration values for micrograms of hydroxyproline per milligram of lipid-free sample are reported.

We injected 1 μl of the derivitized solution on a Luna C18 (2) 3 mm × 75 mm, 3 μm column and used a gradient separation with an Agilent 1100 Liquid Chromatograph with a fluorescence detector to quantify the amount of HYP present. The mobile phase started with a composition of 3% acetic acid (pH 4.3)/acetonitrile 75%/25% and was ramped to 25/75% from 2 to 10 min post-injection at a flow rate of 0.3 ml/min. This composition was maintained for 3 min, after which the column was re-equilibrated for 7 min before the next injection. The fluorenylmethyloxycarbonyl chloride-derivitized HYP and sarcosine were excited at 265 nm, and fluorescence emission was detected at 330 nm. Separation was performed at a column temperature of 40°C. Hydroxyproline eluted at ~7.7 min, whereas sarcosine eluted at 10.3 min. Hydroxyproline values were visually assessed for normality using a Q-Q plot (Dalgaard, 2002), and a summary distribution of values using a box-and-whiskers plot was used to identify extreme outliers. Three values were confirmed as outliers using the Tietjen–Moore test (Tietjen and Moore, 1972) and excluded from subsequent analyses.

### Statistical analyses

Linear regression models were developed to evaluate the relationship of Ps and HYP concentrations in the tissue samples vs. the known ages of the turtles. The effect of subpopulation (each having differing growth rates, age to maturity, reproductive output and activity-season length; J.B.I., unpublished observations) within the study wetlands on the relationship between Ps concentration and age was evaluated using a multivariate regression model, with subpopulation as a class parameter for samples collected across the three panmictic subpopulations (labelled herein as D, M and Z, with the first two from permanent wetlands and the last from an ephemeral wetland).

Decadal means and standard deviations for Ps concentration were also calculated for the entire population, and a one-way ANOVA (followed by a pairwise *t*-test with Bonferroni correction to calculate significance) was performed on the entire data set to determine whether differences were statistically significant. Pairwise *t*-tests were then performed to identify the decadal means that were contributing to the ANOVA result. Means were also compared using a *post hoc* least-significant difference (LSD) test with Bonferroni correction. Statistical analyses were conducted using R (cran-r-project.org) version 3.3.1.

## Results

**Figure 1:**
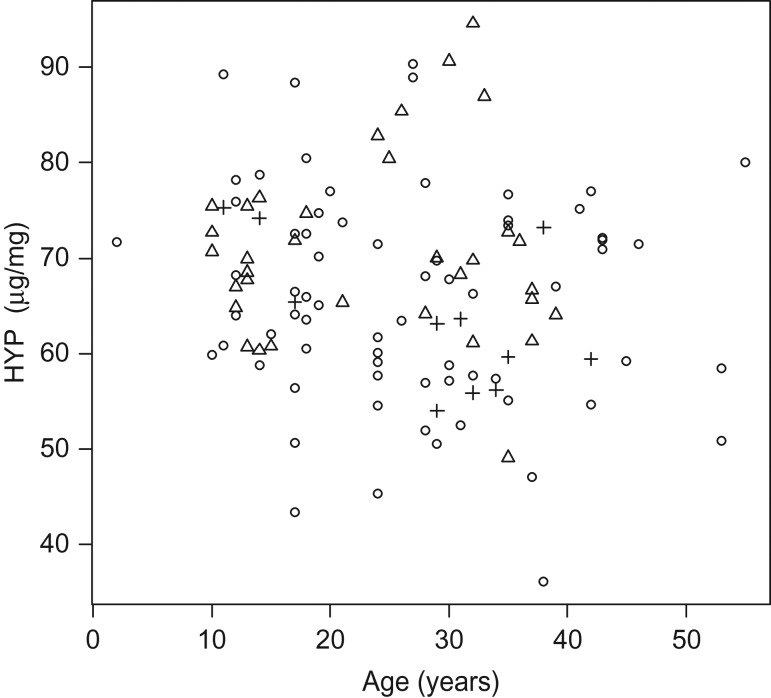
Plot of the hydroxyproline (HYP) concentration (in micrograms per milligram) vs. turtle age for known-age individuals of female yellow mud turtles (*Kinosternon flavescens*) sampled in 2015. The symbols distinguish the different subpopulations, as follows: circles are members of subpopulation D; triangles are from subpopulation M; and plus signs are from subpopulation Z. There is no significant relationship between age and HYP concentration with or without the outliers (see main text).

**Figure 2:**
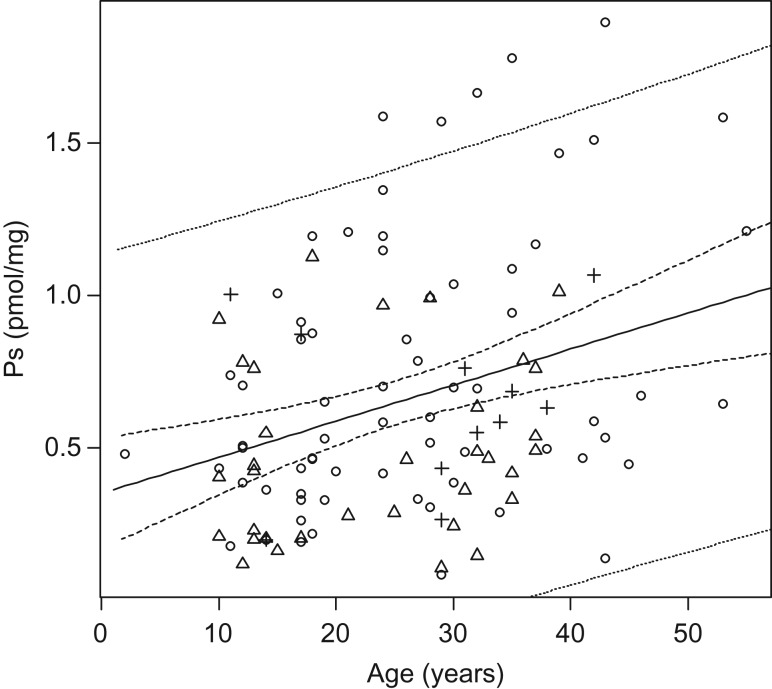
Plot of the pentosidine (Ps) concentration (pmol/mg) vs. turtle age for known-age individuals of yellow mud turtles (*K. flavescens*) sampled in 2015. Subpopulation symbols are as in Fig. 1. The regression equation for all data (see main text) and the 95th percentile confidence interval for the equation (dashed lines) and prediction band for the data set (dotted lines) are also depicted.

**Figure 3:**
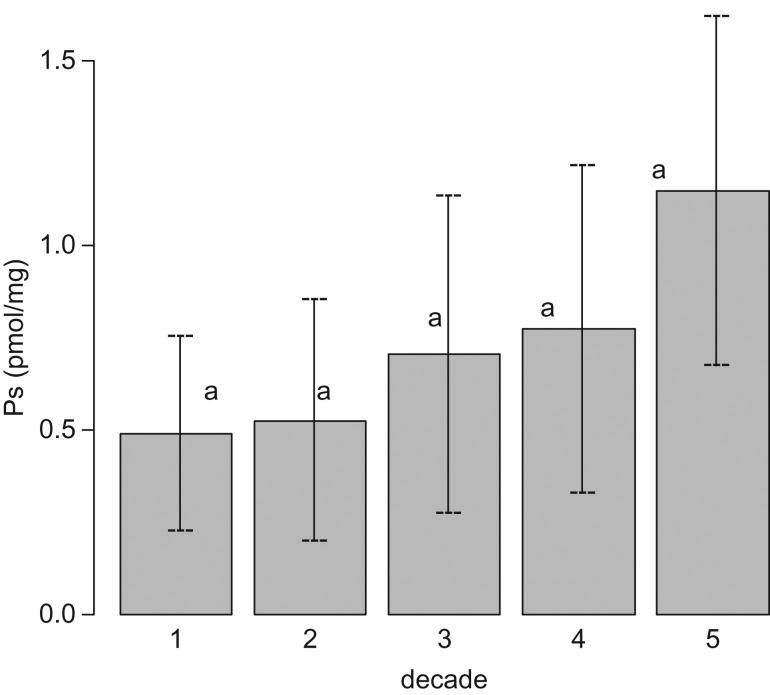
Mean pentosidine (Ps) concentrations (pmol/mg) ± 1 SD plotted by decade of age. Least-significant differences were calculated with a Bonferrroni correction. Lower-case letters denote means that are significantly different at a value of *P* < 0.05.

As expected, the measured HYP concentration (in micrograms per milligram) was not related to age (Fig. 1; HYP = 70.56 − 0.14Age; *R*^*2*^ = 0.021, *F*_1,112_ = 2.36; *P* = 0.13). However, pentosidine concentration (in picomoles per milligram) was positively correlated with age (Fig. 2; Ps = 0.350 + 0.012Age; *R*^*2*^ = 0.105, *F*_1,112_ = 13.08, *P* = 0.0004), although age explained only 10.5% of the variation in Ps concentration. Comparison of decadal mean Ps concentrations (Table 1 and Fig. 3) indicated that the means varied significantly by decade (*F* = 3.45; *P* = 0.011), with a general increase in Ps with decade. However, the pairwise comparison identified few differences between the means for the five decade groups (Table 1), and a LSD test indicated no variation among decadal sample means.

**Table 1:** Comparison of the mean pentosidine concentrations in all the yellow mud turtle samples grouped by decade of age using a pairwise *t*-test; probabilities that means are the same are reported

Decade	*n*	Pentosidine (pmol/mg;mean ± SD)	Pairwise *t*-test comparisons			
			Decade 1	Decade 2	Decade 3	Decade 4
1	5	0.49 ± 0.26				
2	42	0.52 ± 0.33	1.0			
3	29	0.70 ± 0.43	1.0	0.64		
4	38	0.77 ± 0.44	1.0	0.061	1.0	
5	3	1.14 ± 0.47	0.248	0.097	0.669	1.0

The multivariate linear regression model comparing subpopulations captured only slightly more of the variance in the data, with the adjusted *R*^*2*^ = 0.116 (Ps = 0.0112Age − 0.174*popM − 0.116*popZ + 0.44*popD, where the popD [membership = 1; the intercept for the model], popM [membership = 2] and popZ [membership = 3] parameters reflect membership in the respective subpopulation; *F*_3,113_ = 6.06, *P* = 0.0003). The coefficients for Age (*P* < 0.0001), popD (*P* < 0.00001) and popM (*P* < 0.00001) were significant, whereas that for popZ was not (*P* = 0.36).

## Discussion

Considerable research has established the use of pentosidine concentrations as biomarkers for age in birds and mammals. Pentosidine concentrations are in general highly correlated with age in both birds (*r*^2^ = 0.68–0.93; Chaney *et al*., 2003; Fallon *et al*., 2006; Cooey *et al*., 2010; but see Rattiste *et al*., 2015) and mammals (*r*^2^ = 0.57–0.98; Sell *et al*., 1996). However, to our knowledge, changes in pentosidine concentrations with age in an ectothermic vertebrate have not previously been examined. We found a significant but weak correlation between age and pentosodine concentration in the YMT, although wide variation in pentodosine concentrations even in the same decadal age cohort seems to negate the utility of Ps as a precise ageing biomarker, at least in this turtle species. Furthermore, we could demonstrate no significant difference in pentosidine concentrations among our three subpopulations, even though PopZ, which inhabits an ephemeral wetland, exhibits a shorter annual activity season, slower growth and longer age to maturity (J.B.I., unpublished observations) and, presumably, greater physiological stress than the two populations inhabiting permanent waters.

In addition to the variation we documented, the mean Ps concentration of 1.14 ± 0.47 pmol/mg that we report for YMTs >50 years of age is lower than that detected for mature individuals of any endotherm. For example, Sell *et al*. (1996) reported average adult Ps concentrations of 7 pmol/mg in the least shrew (at 3 years of age), 15 pmol/mg in miniature swine (at 14 years), 20 pmol/mg in rhesus monkeys (26 years), 30 pmol/mg in cows (9 years), 60 pmol/mg in dogs (14 years) and ~100 pmol/mg in a human (92 years). They found the rates of Ps formation were greatest in short-lived species and lowest in long-lived species. Their regression equations predicting Ps concentration as a function of age for these species had in all cases, except the least shrew and miniature pig, an intercept predicting a Ps concentration at age = 0 larger than the highest mean concentration at 50+ years of age observed in the YMT.

Ageing processes are complex and often studied in the context of a disease that accelerates the process in mammals. Krivoruchko and Storey (2010) reviewed the sophisticated physiological adaptations expressed in cold-tolerant hibernating turtles and the relevance of these processes in retarding the signs of ageing as expressed in mammals. Pentosidine often accumulates in tissues as a non-enzymatically regulated byproduct of metabolic imbalance in the host (Monnier, 1989; Sell *et al*., 1996). We attempt to provide a contrast between our results and those reported in ageing studies for mammals in the context of various physiological processes that would potentially up-regulate Ps formation in turtles or other species and how those processes are perceived as being down-regulated in turtles.

The low, variable concentrations of Ps in our study could possibly reflect the fact that pentosidine concentrations are known to vary among different tissues in the body of the same animal, at least in endotherms (Holmes *et al*., 2001; Fallon *et al*., 2006), depending on such variables as vascularization, tissue temperatures, collagen turnover rates and antioxidant concentrations. However, we do not believe this serves to explain our data because linear or higher-order relationships between Ps and time still occur in different tissues in endotherms. For example, Cooey *et al*. (2010) documented differences in Ps concentration in skin samples collected from the patagium vs. the breast of vultures and monk parakeets, and they found that Ps concentration in vultures was not different between these two source locations, whereas it was higher in the breast than in the patagium in the monk parakeet. This difference between the two species and sample sites was attributed to differences in flight behaviour, with increased oxidative stress associated with more vigorous flight in the monk parakeet. Oxidative stress significantly increases Ps skin concentration in broiler chicks, which can be offset by feeding haemin, an iron-containing porphyrin (Klandorf *et al*., 2001). The intercepts for the regression equations for Ps concentration in the monk parakeet for both breast and patagium samples calculated by Cooey *et al*. (2010) were also greater than our mean value for the 50+-year-old turtles.

Chaney *et al*. (2003) measured Ps concentrations in the foot webbing of California gulls and determined a linear relationship between Ps concentration and age. The intercept for their equation was 7.47 pmol/mg, again significantly higher than our YMT values. Fallon *et al*. (2006) determined the Ps concentration in museum skin mounts of ruffed grouse and reported 22.5 pmol/mg for a 2-year-old bird. They also examined Ps concentration changes by body location and found that samples from the abdomen had the lowest Ps concentrations (27.5 pmol/mg), whereas the wing had the highest (38.8 pmol/mg). Cooey *et al*. (2010) attributed higher Ps in breast skin than patagium skin to increased vascularization in the former. Given that turtle skin is highly vascularized (Jackson and Ultsch, 2010; Krivoruchko and Storey, 2010), the low Ps concentrations in the YMT are even more striking. Future sampling of other tissues from turtles would be necessary to evaluate whether intra-individual variation in Ps concentrations is important, although other tissues will be much more difficult to sample without negative impact to the turtle.

Sell *et al*. (1996) demonstrated that the interaction of metabolism and longevity in six mammalian species was correlated with Ps accumulation in skin tissues, and Cerami (1985) proposed that glucose was a mediator in the ageing process in mammals. As Ps is a glycolytic byproduct, the extremely low concentrations of Ps detected in the skin samples of YMTs probably reflect the lower metabolic rate of this poikilotherm, the metabolic depression that occurs as a result of extreme cold exposure, the adaptive mechanisms that reduce formation of reactive oxygen species during hibernation and the physiological mechanisms that allow for anoxia while diving (each discussed below). The initial physiological responses of numerous chelonian species to these stressors have been demonstrated to increase glucose and some amino-acid concentrations in blood (Reese *et al*., 2001; Storey, 2006; Warren and Jackson, 2007; Costanzo and Lee, 2013), and this favours Ps formation.

Our YMT population inhabits a region of temperature extremes and exhibits one of the shortest annual activity seasons of any turtle (generally <128 days; Christiansen *et al*., 1985). Hence, we associate the high variance that we observed in our samples with the complex interaction of climate, growth rate, age, duration of hibernation and individual exposure to stress during hibernation; however, insufficient details of these life-history parameters exist for our individuals to allow us to evaluate their effects on our Ps data.

Pentosidine accumulation is greater in conditions of oxidative stress (Fallon *et al*., 2006; Rattiste *et al*., 2015), but turtles are relatively resistant to this stress at lower temperatures (Baker *et al*., 2007; Treidel, 2015). Also, in endotherms, hyperglycaemia generally increases Ps accumulation (Dyer *et al*., 1991; Mikulíková *et al.,* 2008), and hyperglycaemia in turtles occurs in response to hibernation or anoxia (Jackson and Ultsch, 2010), but this apparently does not result in increased Ps in the skin of the YMT. Furthermore, one might expect reduced oxygen delivery to the skin in cold and/or anoxic conditions as a result of reduced capillary circulation, limiting Ps accumulation.

Terrestrially dormant (both aestivating and hibernating) YMTs have been demonstrated to incur an oxygen deficit based on increased respiratory levels (Seidel, 1978) and elevated plasma lactate concentrations (Chilian, 1976), which are both associated with the anaerobic metabolism reported in other cold-tolerant, hibernating turtles (Jackson and Ultsch, 2010; Costanzo and Lee, 2013). Yellow mud turtles do not exhibit the freeze tolerance observed in hatchlings of *Chrysemys picta* or *Terrapene ornata* (Costanzo *et al*., 1995), and at our study site, adult and hatchling YMTs hibernate in terrestrial burrows below the frost line (Iverson, 1991; Iverson *et al*., 2009) and, presumably, do not experience the physiological demands associated with the anoxia/hypoxia endured by turtles that hibernate underwater. Nevertheless, hibernating turtles maintain elevated concentrations of blood glucose to maintain brain and heart function (Jackson and Ultsch, 2010), and elevated glucose concentrations contribute to increased rates of Ps formation in endotherms (e.g. Dyer *et al*., 1991; Mikulíková *et al*., 2008). In humans, Ps and other advanced glycation end products contribute to oxidative stress in diabetics (i.e. in hyperglycaemic conditions; Nowotny *et al*., 2015). But turtles have been found to mount significant antioxidant defenses to protect against reactive oxygen species produced as a result of metabolic shifts associated with anoxia and hibernation (Krivoruchko and Storey, 2010), and these responses may have led to the reduced Ps concentrations detected in the present study.

Even so, the results of this study were surprising to us, given the wide age spread of the turtles and the bouts of hyperglycaemia they would experience at least annually. What we had not anticipated was the unique physiological adaptations that turtles have that serve to ameliorate physiological stress resulting from exposure to anoxia during diving or hibernation and the degree to which these adaptations suppress Ps formation. For example, in turtles (and other vertebrates) oxidative stress significantly up-regulates the formation of α-oxyaldehydes (Abordo *et al*., 1999), which are cytotoxic and predispose tissues to the formation of Ps cross-links (Aćimović *et al*., 2014). These α-oxyaldehydes are detoxified via a glutathione-dependent glyoxylase system, and the concentrations of glutathione in turtle organs are higher than those observed in other ectotherms (Willmore and Storey, 1997). Glutathione (see above) inhibits pentosidine formation (Labuza *et al*., 1994). Additionally turtle glutathione reductase has a higher affinity for substrate across a broader pH range than is observed in other species (Willmore and Storey, 2007), offsetting the effects of acidosis on this pathway as a result of lactic acid accumulation observed during hibernation (Hochachka, 1986; Churchill and Storey, 1992; Packard and Packard, 2004, 2005; Jackson and Ultsch, 2010).

Furthermore, concentrations of the antioxidant ascorbic acid (ascorbate) have been found to be relatively high in the central nervous system of turtles, but to vary positively with temperature (Pérez-Pinzón and Rice, 1995) and to be highest in turtles that are anoxia tolerant (Pérez-Pinzón and Rice, 1995). Although ascorbate concentrations are in general positively correlated with pentosidine production (at least in mammals, e.g. Nagaraj *et al*., 1994), ascorbate concentrations are tissue specific (declining peripheral to the brain itself) and also tightly regulated seasonally (Pérez-Pinzón and Rice, 1995). In any case, as noted above, the YMT hibernates terrestrially and, presumably, does not exhibit elevated ascorbate concentrations, although they have not been quantified in this species. Hence, they may be irrelevant to Ps production in this species.

These adaptations appear to be specific to turtles that are cold tolerant, anoxia tolerant and hibernate, and they are likely to contribute significantly to the low concentrations of Ps detected in the present study. Therefore, in YMTs, extreme temperature variation in the environment, moderated by physiological adaptations, may drive variation in pentosidine concentrations more than does ageing. The relative contributions of hibernation vs. diving adaptations in lowering Ps accumulation might be teased apart in temperate-zone turtles by investigating Ps patterns in other terrestrial species (such as *Terrapene*) that hibernate but do not engage in diving.

If physiological adaptations for prolonged exposure to anoxic conditions overwhelm the signature of ageing in Ps accumulation for turtles from cold-temperate regions, it may be that species from lower latitudes with longer activity cycles and no need to hibernate may have different patterns of pentosidine accumulation. We anticipate that given the absence of these adaptations in tropical ectotherms, it might be possible to use Ps as a biomarker to age them. We suggest that this would be a worthy object of investigation, because most of the world's threatened turtles are from subtropical or tropical regions (Turtle Taxonomy Working Group, 2014), and having a reliable ageing biomarker could, as stated earlier, prove invaluable in assessing the demographic health of these populations and for estimating the potential reproductive senescence of captive animals of unknown provenance. Unfortunately, we are unaware of any long-term field studies of tropical turtles (including marine turtles) that could provide tissue samples from animals of known ages.
